# The Value of In Vitro Tests to DiminishDrug Challenges

**DOI:** 10.3390/ijms18061222

**Published:** 2017-06-07

**Authors:** Cristobalina Mayorga, Inmaculada Doña, Ezequiel Perez-Inestrosa, Tahia D. Fernández, Maria J. Torres

**Affiliations:** 1 Research Laboratory-Allergy Unit, Biomedical Institute of Málaga-IBIMA, Regional University Hospital of Malaga-UMA, Málaga 29009, Spain; tahiadfd@gmail.com; 2 Allergy Service, IBIMA-Regional University Hospital of Malaga-UMA, Málaga 29009, Spain; inmadd@hotmail.com (I.D.); mjtorresj@gmail.com (M.J.T.); 3 Department of Organic Chemistry, University of Málaga, Biomedical Institute of Málaga-IBIMA, Málaga 29071, Spain; inestrosa@uma.es; 4 Andalusian Center for Nanomedicine and Biotechnology-BIONAND, Málaga 29590, Spain

**Keywords:** drug, hypersensitivity, allergy, diagnosis, in vitro, IgE, T-cells, basophils, cytokines, immunoassays

## Abstract

Drug hypersensitivity reactions have multiple implications for patient safety and health system costs, thus it is important to perform an accurate diagnosis. The diagnostic procedure includes a detailed clinical history, often unreliable; followed by skin tests, sometimes with low sensitivity or unavailable; and drug provocation testing, which is not risk-free for the patient, especially in severe reactions. In vitro tests could help to identify correctly the responsible agent, thus improving the diagnosis of these reactions, helping the physician to find safe alternatives, and reducing the need to perform drug provocation testing. However, it is necessary to confirm the sensitivity, specificity, negative and positive predictive values for these in vitro tests to enable their implementation in clinical practice. In this review, we have analyzed these parameters from different studies that have used in vitro test for evaluating drug hypersensitivity reactions and estimated the added value of these tests to the in vivo diagnosis.

## 1. Introduction

Drug hypersensitivity reactions (DHR) represent 5–10% of all adverse drug reactions [1]. Longer inpatient stays and higher rates of hospital associated infections have been reported for antibiotic allergic patients [2]. These reactions have multiple implications for patient safety and health system costs, often requiring alternative drugs to be prescribed; these alternative drugs may be less effective, more toxic and more expensive; moreover, in the case of antibiotics, this can augment the development of bacterial resistance [2]. For these reasons, it is important to establish an accurate diagnosis of DHRs, and to avoid labeling tolerant individuals as allergic. However, it is just as important to correctly identify the responsible agent and find safe alternatives to avoid serious problems due to reactions. This is particularly important for severe DHR such as anaphylaxis, Steven–Johnson Syndrome (SJS) and Toxic Epidermal Necrolysis (TEN).

Although in theory drugs can induce the four types of reaction proposed in the Coombs and Gell classification [3], types I and IV are the most frequent. Type I or immediate DHR (IDHR) are mediated by drug specific IgE (sIgE) antibodies attached to high-affinity IgE receptors, FcεRI, on mast cells or basophils, inducing release of mediators that lead to the reaction [3]. Type IV or non-immediate DHR (NIDHR), are induced by T-cells through the involvement of different inflammatory mediators [4]. Different reaction types show different clinical manifestations and timings, therefore mechanism should be taken into account during the allergological work-up.

The diagnostic procedure of a suspected DHR includes a detailed clinical history [5], followed by skin tests (STs) [6] and drug provocation testing (DPT) [7]. This procedure can be complex, time-consuming, and expensive. Moreover, it can present some risk to the patient. A detailed clinical history is the most important step towards an accurate diagnosis of DHR. However, it can be unreliable since there may be a lack of accurate information, i.e., the chronology may be imprecise, the clinical manifestations may be heterogeneous and the exact name of drug or corrective treatment may be not recalled precisely by the patient, making drug causality assessment difficult to ascertain [8].

Regarding STs, their diagnostic value is not well established for most drugs. Detailed, validated ST protocols for the diagnosis of DHR are lacking, and test concentrations are unknown or poorly validated. In many cases, STs have low sensitivity and require high drug concentrations; this can result in false-positive reactions due to the irritative properties of the drug. Moreover, many drugs are not available in injectable form and hence intradermal tests are not possible. Although STs are not validated and standardized for all drugs [6,9], experts from both Europe and America suggest that it is possible to recommend specific drug concentrations for β-lactam (BLs) antibiotics, perioperative drugs, heparins, platinum salts, and radio contrast media (RCM) [9].

Since clinical history can be unreliable and the sensitivity of STs may be suboptimal or unknown, the definitive diagnosis of DHR frequently relies upon DPT [10]. DPT must be performed by controlled administration under medical surveillance. It is widely considered to be the gold standard to establish or exclude the diagnosis of hypersensitivity to a certain substance. It not only reproduces allergic symptoms but also any other adverse clinical manifestations, irrespective of the mechanism. Moreover, it can be used to provide alternative drugs [10]. However, DPT is a procedure that consumes time and resources and, due to the possibility of reproducing the allergic reaction, is not risk free, especially when evaluating severe reactions. Therefore, it should be performed after balancing the risk–benefit ratio for each individual case. Patients at risk of more severe reactions should be given DPT in a hospital setting. It should not be performed in patients with co-morbidities such as acute infections or serious underlying diseases, as drug-exposure might provoke reactions that are hard to control. It should not be performed in patients who have experienced severe life-threatening reactions such as anaphylaxis, SJS or TEN [10].

Given the low sensitivity of in vivo procedures, potential unreliability of clinical history, and riskiness of DPT, there is a clear need for the development of validated in vitro tests to aid and improve the diagnosis of DHR. These tests will help us to correctly identify the responsible agent for a reaction and evaluate cross-reactivity with other drugs, helping the physician to find safe alternatives, and reducing the need to perform DPT.

## 2. In Vitro Tests for the Diagnosis of Drug Hypersensitivity Reactions (DHR)

Nowadays, in vitro methods for evaluating DHR depend on the underlying mechanism, whether IgE or T cell-mediated, and are mainly based on the analysis of drug sIgE or T cell subpopulations as well as the detection of specific markers after stimulation with the culprit drug and/or its metabolites.

As noted above, IDHR are IgE mediated. Therefore, the most widely used in vitro tests are immunoassays and basophil activation test (BAT). For NIDHR, which are cell-mediated, lymphocyte transformation tests (LTT), enzyme-linked immunosorbent spot assay (ELISpot), enzyme-linked immunosorbent assay (ELISA) and flow cytometry are typically used [11,12]. However, there is high variability between published studies regarding their accuracy. This is likely due to small sample sizes, and in some studies the lack of appropriate control subjects. There is no general consensus regarding under which circumstances these tests are most appropriate. Recently, experts from the European Network on Drug Allergy and Drug Allergy Interest Group of the European Academy of Allergy and Clinical Immunology provided recommendations regarding the available in vitro tests for DHR. They identified several unmet needs from which they highlighted the necessity to confirm the sensitivity, specificity, NPV and PPV for these in vitro tests. Such data are crucial to enable the implementation of in vitro testing in clinical practice, in order to reduce the need to perform DPT.

In this review, we have analyzed sensitivity and specificity as well as positive predictive value (PPV) and negative predictive value (NPV). Where possible, we have calculated these values using the data given in the studies. In total, we have obtained values from 60 publications, selecting studies that used in vitro test for evaluating DHR, for at least five patients. We have also estimated the added value of these tests to the in vivo diagnosis, paying special attention to how they can lead to increased ST sensitivity, avoiding the need to perform DPT.

## 3. IgE-Mediated Reactions

The main goal of in vitro tests for these types of reaction is the determination of drug/drug metabolite sIgE, either soluble in serum, or bound to the basophil surface. However, since sIgE and especially drug sIgE are found at a very low concentration in the blood [13], these in vitro methods must be highly sensitive. Another important issue is the need for different drugs to bind covalently to a carrier protein such as a hapten; this is required for a drug to induce an immune response. It is therefore important that the test includes the correct carrier molecules for appropriate IgE recognition [14,15]. All these factors are critical for in vitro test development.

The most frequent methods to evaluate IDHR are immunoassays (radioimmunoassays and fluorimmunoassays) and tests based on basophil activation (BAT).

### 3.1. Immunoassays

“Immunoassays” refers to a group of techniques that are based on the quantification of drug-sIgE present in patient sera. For this, the drug is conjugated to a carrier and coupled to a solid phase which is incubated with patient serum. If present, sIgE in the sera recognizes the drug, forming a drug-carrier-antibody complex which is quantified using a secondary anti-human IgE antibody labeled with a radioisotope (RIA) or a fluorescent enzyme (FEIA) [11]. RIA is generally conducted using in house techniques, such as the radioallergosorbent test (RAST); FEIA can be performed using commercial products, such as the ImmunoCAP-FEIA, although such products are only available for few drugs [16,17].

The majority of studies for evaluating the value of immunoassays for diagnosing IDHR have been performed with BLs [13,15,16,18,19,20,21,22] and neuromuscular blocking agents (NMBAs) [23,24,25,26,27,28,29], although a handful of studies have been carried out on other drugs, including chlorhexidine [30], quinolones [31,32] and biological agents [33,34,35,36]. Many of these studies have included large numbers of allergic patients and controls, allowing us to estimate the sensitivity, specificity, PPV and NPV (Table 1). Overall, we have calculated an average sensitivity of 62.9%, specificity of 89.2% and a PPV and NPV of 83.3% and 77.8%, respectively. In our opinion this sensitivity value is sub-optimal for clinical diagnosis, especially for BLs and no-BLs antibiotics. Moreover, NPV, the most useful parameter to decide whether to perform a DPT, is also relatively low [37]. Interestingly, immunoassays show higher results for NMBAs, with sensitivity of 79.3%, specificity of 92.2% and PPV and NPV of 91.3% and 83.3%, respectively. Regarding biological agents, although the global sensitivity is low (48.2%), immunoassays show a high specificity (92.5%) and NPV (83.6%).

### 3.2. Basophil Activation Test

This test is based on the determination of basophil activation or degranulation markers after drug stimulation using flow cytometry [17,38]. Compared with the determination of sIgE by immunoassays, BAT analysis is used to demonstrate a functional response [37]. BAT has been shown to be useful as an additional test for the diagnosis of DHR, especially for those cases where there are no other diagnostic tool available besides DPT. Basophils can be detected using a single cell marker, such as anti-IgE, CCR3, CRTH2, or CD203c, or a combination of several. Activation is usually assessed by determining the expression of CD63 or CD203c on the basophil surface [37]. It is important to take into account that differences have been found in the upregulation of both markers depending on the drug tested and the clinical entity [39,40]. When evaluating DHR using BAT, the possibility of activating basophils by a non-IgE-mediated mechanism exist, thus the involvement of the FcεRI-mediated pathway should be confirmed by inhibition with PI3Kinase inhibitors such as wortmannin [32,41].

As for immunoassays, multiple studies with BAT using reasonable numbers of patients have been performed for BLs [18,19,39,42,43,44,45] and NMBAs [24,25,46,47,48,49,50,51]. Additional studies have been performed for fluoroquinolones (FQs) [31,40,52,53], pyrazolones [54,55,56] and RCM [57,58] (Table 2). The analyses of these studies, taken together, show an average sensitivity of 59.4%, specificity of 94.6% and PPV and NPV of 93.4% and 66.3%, respectively. Sensitivity and specificity are relatively similar for the different drugs, ranging from 51.7% to 66.9% and 89.2% to 97.8%, respectively. The lowest values of sensitivity and NPV have been found for BLs, being 51.7% and 49.9%, respectively. On the contrary, the highest sensitivity and NPV values were found for IDHR to FQs, 64.4% and 68.1%, respectively, and NMBAs, 66.9% and 72.1% respectively.

### 3.3. Combining In Vitro Tests for Evaluating Immediate Drug Hypersensitivity Reactions (IDHR)

**Figure 1:**
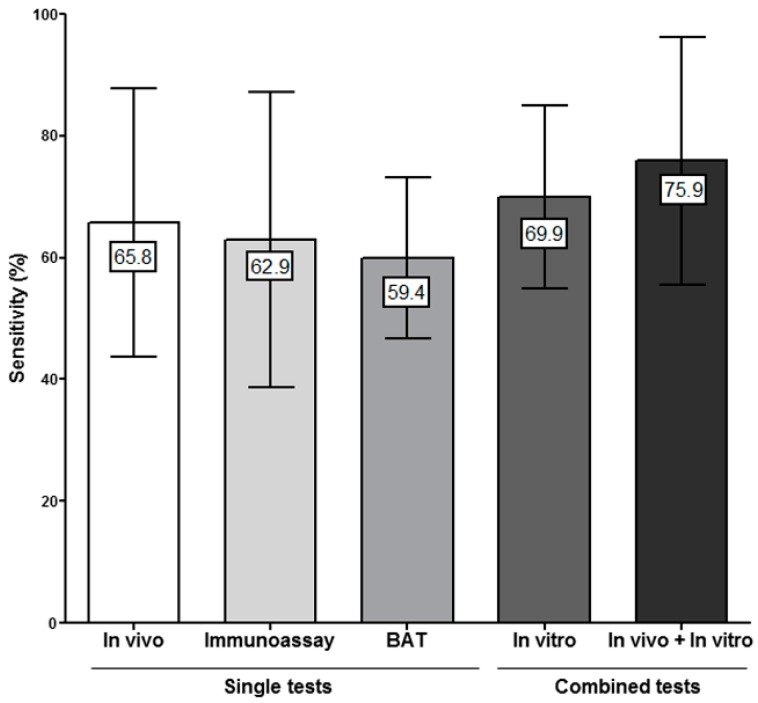
Bars represent the global sensitivity (mean plus standard deviation) for in vivo and in vitro tests alone or in combination for the evaluation of immediate drug hypersensitivity reactions (IDHR). BAT: Basophil activation test.

**Figure 2:**
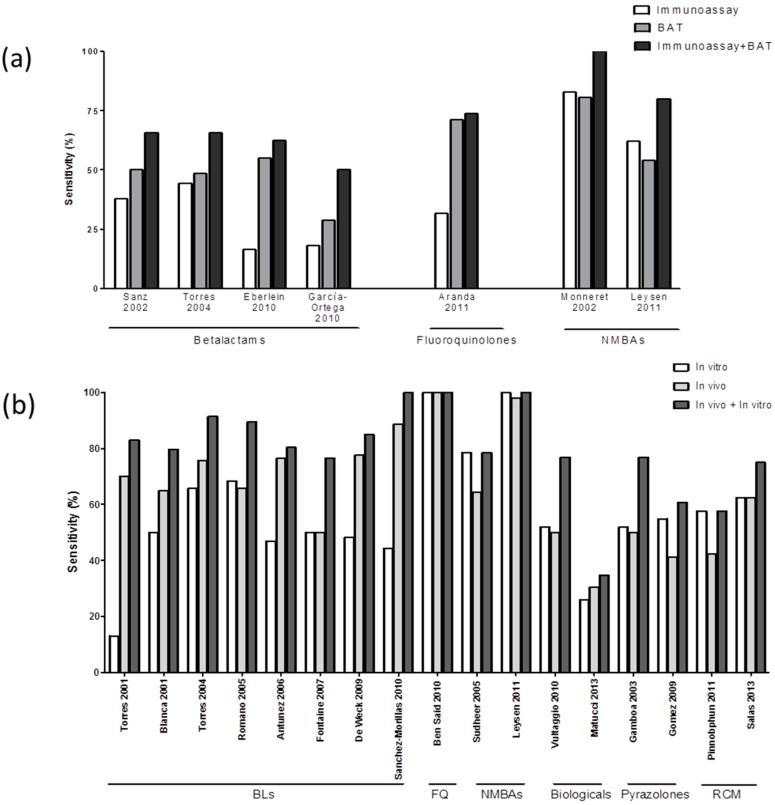
(**a**) Bars represent the sensitivity of immunoassays (**white bars**), basophil activation test (BAT) (**light grey bars**) and immunoassay + BAT (**dark grey bars**) in individual studies performed with patients with IDHR to BLs, FQs or NMBAs; and (**b**) Bars represent the sensitivity of in vitro tests (including immunoassay and BAT) (**white bars**), in vivo tests (**light grey bars**) and combination of the results of both in vitro and in vivo tests (**dark grey bars**) in individual studies performed with patients with IDHR to different drug groups. BLs: β-lactams; FQ: Fluoroquinolones; NMBAs: Neuromuscular blocking agents; and RCM: Radio contrast media.

Several studies have performed both tests, immunoassays and BAT, for BLs [18,42,44,59], FQs [31] and NMBAs [24,25]. We have used the data from these studies to estimate the diagnostic value obtained by combining the results of both tests. We found an overall mean in vitro sensitivity of 69.9% (Figure 1). Combining immunoassay + BAT results according to the culprit drug showed an increase in sensitivity of around 20%, for both BLs and NMBAs, compared to using only one test. In the only study performed in FQs, the inclusion of RIA had little effect compared to the results obtained with BAT alone (Figure 2a). The specificity of the combined in vitro tests was 90.9% which is in the range of the mean specificity of individual tests (89.2–94.6%).

### 3.4. Combining In Vitro and In Vivo Tests for Evaluating IDHR

We evaluated the sensitivity obtained when combining the results of both in vivo and in vitro tests, using studies that included ST data. We observed an increase from 65.7% when only in vivo tests are considered, to 75.9% when including both in vitro and in vivo tests (Figure 1). Combining in vivo and in vitro testing lead to an average increase of around 15% in sensitivity over in vivo testing alone for eight studies performed in BLs allergic patients [16,19,20,42,45,60,61,62] as well as for other drugs such as NMBAs [24,48], biological agents [34,36], pyrazolones [55,56] or RCM [57,58] (Figure 1 and Figure 2b). Regarding specificity, it was 91.8%.

It is interesting to note that in vitro tests, such as BAT, can produce positive results in IDHR patients who give negative STs; in fact 40% of ST negative patients with IDHR to BLs give positive BAT results [19,42]; this figure is around 12% for FQs [53] and 30% for pyrazolones [55,56]. These results clearly indicate that the inclusion of in vitro tests complement the results of in vivo testing in the evaluation of IDHR, increasing the diagnostic sensitivity [24]. However, we must take into account that in this test we can found 1–10% false positive results.

## 4. T Cell-Mediated Reactions

The evaluation of cell-mediated DHR or NIDHR is more complex than for IDHR, mainly due to the heterogeneity of clinical symptoms. These differences in symptoms imply that although in most reactions T cells are involved, many other cell subpopulations are likely to play a critical role [41,63]. It is therefore important to study and characterize effector cells and their corresponding inflammatory mediators.

Most of the in vitro tests used for evaluating NIDHR have as their main goal the assessment of the drug involved in the reaction. The idea is to reproduce the effector reaction in vitro by activating T cells and inducing the appropriate inflammatory and cytotoxic mediator release that will be determined by LTT, ELISpot or ELISA. Most currently available studies for evaluating these types of reactions include small numbers of patients and have included a heterogeneous mix of patients and culprit drugs, generally including BLs, anticonvulsants, local anesthetic and NSAIDs. Another important limitation of these studies is that in most publications, the diagnosis has been confirmed by clinical history and/or STs and did not include DPT. Therefore, we cannot know if a patient would react to the drug. This limitation is compounded by the low sensitivity of STs in this type of reaction [64].

### 4.1. Lymphocyte Transformation Test (LTT)

This test is based on the determination of the lymphocyte proliferative response after stimulation with the specific drug [12]. This proliferation has classically been measured via the incorporation of tritiated thymidine (^3^H) into the genome of proliferating cells and assessed by measuring the increase in radioactivity in a liquid scintillation counter system [38]. With advances in flow cytometry, the possibility of assessing proliferation by the serial dilution of a fluorescent molecule (carboxyfluorescein diacetate succinimidyl ester (CFSE)) into the cells has appeared, introducing the possibility of identifying the effector cells involved in the reaction [11]. However, there is a lack of studies comparing these two methods in terms of sensitivity and clinical value [17].

We have analyzed the results of 13 studies [65,66,67,68,69,70,71,72,73,74,75,76,77] of LTT containing at least five patients. We calculated a mean sensitivity of 56.1% and a specificity of 93.9%, while PPV and NPV were 92.3% and 63.2% respectively (Table 3). Higher values were found when evaluating studies including mild and moderate reactions [65,66,67,68,69,70,71,72], showing sensitivity and specificity values of 65.1% and 96.5% with PPV and NPV of 94.4% and 67.2%, respectively. However, these figures decreased when we only included severe reactions [73,74,75,76,77], showing a sensitivity of 39.9%, specificity of 89.8% and PPV and NPV of 87.8% and 52.4%, respectively. These data suggest that LTT is better suited to evaluating moderate NIDHR compared to severe reactions, such as TEN and organ specific reactions [11,12,75].

Moreover, some studies suggest that the sensitivity of the LTT for the diagnosis of NIDHR can be improved by including dendritic cells as antigen presenting cells [56,68,78] as well as other innate factors that where involved in the original reaction [79].

### 4.2. Enzyme-Linked Immunosorbent Spot (ELISpot)

ELISpot determines the number of cells producing an inflammatory marker, such as relevant cytokines and cytotoxic markers, after their activation by the specific drug [80]. This method provides both qualitative and quantitative information and has demonstrated to be highly sensitive, enabling the detection of less than 25 secreting cells per 10^6^ peripheral blood mononuclear cells [17,38]. It has been increasingly used for the evaluation of effector cells in NIDHR over the last decades [67,74,77,81,82,83,84]. The six studies using ELISpot that have been included in this review are focused on BLs and/or anticonvulsants and measure the number of cells producing IFN-γ, IL-4, IL-5 or Granzyme B [67,76,77,85,86,87]. Data showed a mean sensitivity of 61.2%, a specificity of 98.6, a PPV of 96.2% and a NPV of 59.1% (Table 3).

### 4.3. Flow Cytometry and Enzyme-Linked Immunosorbent Assay (ELISA)

Another approach for evaluating NIDHR after stimulation with the specific drug is by the determination of the cell activation and/or cytokines or cytotoxic production by either flow cytometry analysis of cells in culture or ELISA using the culture supernatants [72,79,88,89,90,91,92]. These methodologies have been used for determining IL-2, IL-5, IL-10 and IFN-γ [74,93]. We have included four studies that contained over five patients [74,93,94,95], which found the mean sensitivity of these tests to be 66.6% and the specificity 87.5%, with PPV and NPV being 88.5% and 69.5%, respectively (Table 3).

### 4.4. Combining In Vitro Tests for Evaluating Non-Immediate Drug Hypersensitivity Reactions (NIDHR)

**Figure 3:**
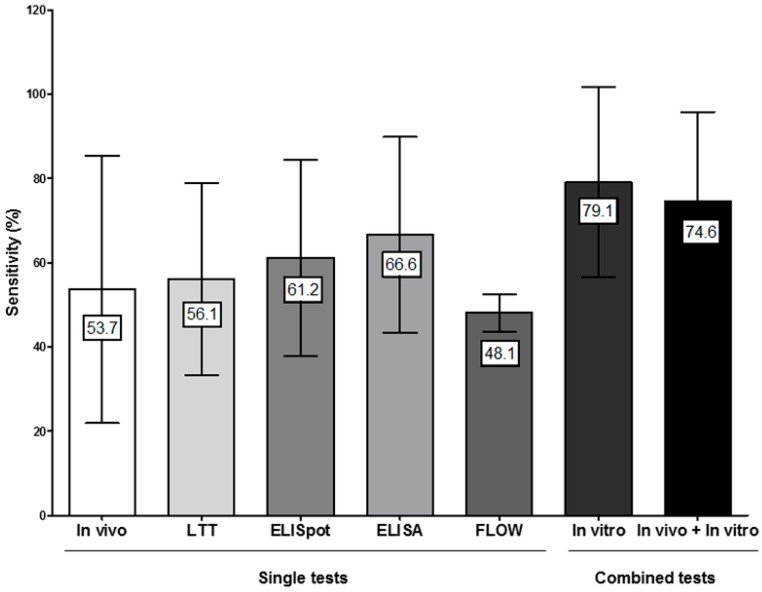
Bars represent the global sensitivity of different in vivo and in vitro tests (mean plus standard deviation), either alone or in combination, for the evaluation of non-immediate drug hypersensitivity reactions (NIDHR).

**Figure 4:**
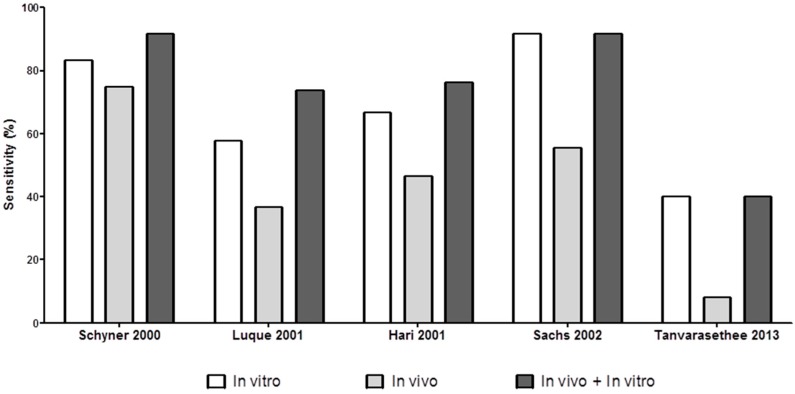
Bars represent the sensitivity of in vitro tests (**white bars**), in vivo tests (**light grey bars**) and combination of the results of both in vitro and in vivo tests (**dark grey bars**) in individual studies performed with NIDHR patients.

Although the sensitivity of the above-mentioned tests for evaluating NIDHR showed a similar values ranging from 56.1 to 66.6% (Table 3), it has been proposed that the combination of results from different assays could be useful [76,77,93]. Different combinations have been reported, including LTT and a panel of cytokines/cytotoxic molecules determined by ELISpot, flow cytometry and/or ELISA (using IFN-γ, IL-2, IL-4, IL-5, granzyme B and granulysin) [67,74,76,77,85,93]. Here, we have calculated the sensitivity of the combination of the results from different tests including data from those studies that performed at least two different in vitro methods. Results show an increase in sensitivity, up to 79.1% when evaluating patients with both moderate and severe NIDHR to betalactams and anticonvulsants (Figure 3 and Figure 4). The specificity of the combined in vitro tests was 97.5% which is in the range of the specificity of individual tests (87.7–98.6%) (Table 3).

These data indicate that the combination of different in vitro tests could help better identify the culprit drug in these types of reactions.

### 4.5. Combining In Vitro and In Vivo Tests for Evaluating NIDHR

We further analyzed the sensitivity of the allergological evaluation of NIDHR when the results of in vitro tests were combined with those obtained in in vivo (STs) tests and observed that it increases from 53.7% to 74.6% (Figure 3 and Figure 4). This is very important if we take into account that many of these reactions show severe symptoms and that in many cases in vivo testing cannot be performed.

Interestingly, the mean sensitivity of in vitro tests (79.1%) is higher than those we have obtained when combining in vivo and in vitro results (74.6%), this is because different studies have been included in each case. Most studies that included in vivo sensitivity data used LTT as the in vitro test, which is relatively less sensitive. Thus, more studies including either ELISpot or ELISA/Flow cytometry in combination with skin testing should be performed in order to obtain a more accurate estimate of sensitivity for the in vivo/in vitro evaluation of NIDHR. Regarding specificity, it was 97.8%.

## 5. Conclusions

DHR diagnosis is a complex and unresolved issue due to the low sensitivity of the different approaches and the possible risks associated with performing DPT, especially for severe reactions, both for IDHR and NIDHR. The data presented in this review indicate that current in vitro methods, although not sufficient by themselves, can be helpful in assessing IDHR and NIDHR, and have been shown to increase the overall sensitivity of the diagnostic procedure when combined with in vivo testing.

They show, in general, a low sensitivity that depends on the drug involved and for those evaluating NIDHR also on the clinical manifestation. The low sensitivity could be related to other factors such as the use of non-appropriate drug metabolite(s) or drug-carrier conjugates that hide the relevant epitopes.

Cellular test has shown similar values of PPV and NPV independently of the type of reaction, IDHR or NIDHR. Additionally, it is important to note that, although NPVs are not optimal, in vitro tests have shown a good specificity, which correlates with a high PPV indicating that they could reduce the number of false positive results and help to decrease the administration of alternative drugs that, as mentioned before, could be less effective and induce undesired side effects. On the other hand, the low sensitivity could have serious effects when falsely labeling a patient as non-allergic, especially for severe reactions.

There is certainly room for improvement in in vitro testing. Areas requiring attention include: characterizing the drug metabolites involved in the reactions and that are recognized by the immunological system; characterizing the effector immunological mechanism involved in order to determine specific biomarkers; and combining results from multiple in vitro and in vivo tests.

Further studies are needed that include a large number of patients and controls and that take into account the combination of in vivo and in vitro tests in order to evaluate the real added value of the latter and therefore the possibility of avoiding the performance of drug provocation tests.

## Figures and Tables

**Table 1:** Immunoassays in immediate reactions to different drugs.

Paper	Patients	Drugs	Diag	Method	Sens (%)	Spec (%)	NPV (%)	PPV (%)
**Betalactams**								
Garcia 1997 [15]	30 Pat 30 Cont	PG	ST	RAST	86.66	90	87.09	89.65
Blanca 2001 [16]	74 Pat 55 Cont	AX, PG	ST, DPT	CAP	50	96	58.8	94.4
Sanz 2002 [18]	58 Pat 30 Cont	PG, AX, AMP, CEFU, CEFAZ	ST	CAP	37.9	86.7	41.9	84.6
Garcia-Aviles 2005 [21]	67 Pat 30 Cont	AX, PG CEFU, CEPHA	ST, DPT	CAP	37.8	83.3	37.5	83.5
Fontaine 2007 [20]	30 Pat 15 Cont	AX, PG, AMP, CEFOT, CEFT, CEPH, CEFAC	ST, DPT	RAST	50	73.3	42.3	78.9
				CAP	16.6	93.3	35.9	83.2
De Weck 2009 [19]	178 Pat 81 Cont	BP, AX, AMP, CEFs	ST, DPT	CAP	28.3	86.5	35.4	82.1
Vultaggio 2009 [13]	61 Pat 115 Cont	PG, PV, AX, AMP	ST	CAP	85	54	87.2	49.5
Vultaggio 2015 [22]	171 Pat 122 Cont	PG, PV, AX, AMP	ST	CAP	66	52	52.2	65.8
				CAP	43	95	54.3	92.3
**Mean Values**					**50.1**	**81.01**	**53.3**	**80.4**
**SD**					**23.0**	**16.2**	**19.6**	**13.5**
**Quinolones**								
Manfredi 2004 [32]	55 Pat 32 Cont	CIPRO, LOME, NORFL, OFLO, PIP, RUFL, PEFL, NALI	CH	SEPH	54.5	100	56.1	100
Aranda 2011 [31]	38 Pat 25 Cont	CIPRO, MOXI, LEVO	CH, DPT	SEPH	31.6	100	49.0	100
**Mean Values**					**43.1**	**100**	**52.6**	**100**
**SD**					**16.19**	**0**	**5.0**	**0**
**NMBAs**								
Guilloux 1992 [26]	31 Pat 34 Cont	MOR, SUC, ALCU, TMA, TEA	ST	RIA	96.7	97.2	96.9	96.9
Mata 1992 [28]	40 Pat 44 Cont	SUX, VECU, PANCU, ALCU, ATRAC, GALLA	CH, ST	SEPH	82.5	100	86.2	100
Monneret 2002 [25]	39 Pat 17 Cont	ROC, SUX, ATRAC	CH, ST	RIA	62	100	53.4	100
Ebo 2007 [23]	25 Pat 30 Cont	ROC, SUX, MOR, PHO	ST	CAP	ROC: 92	ROC: 93	ROC: 93.3	ROC: 92
					SUX: 72	SUX: 100	SUX: 81.1	SUX: 100
					MOR: 88	MOR: 100	MOR: 90.9	MOR: 100
					PHO: 86	PHO: 100	PHO: 89.5	PHO: 100
Leysen 2011 [24]	41 Pat 25 Cont	ROC	CH, ST	CAP	82.9	72.0	72.1	82.9
Laroche 2011 [29]	57 Pat 54 Cont	MOR	CH, ST	CAP	84.2	90.7	84.2	90.7
Rouzaire 2012 [27]	11 Pat 20 Cont	ROC, SUX, MOR	CH, ST	CAP	SUX: 44	SUX: 100	SUX: 76.4	SUX: 100
					ROC: 83	ROC: 68	ROC: 87.9	ROC: 59
					MOR: 78	MOR: 85	MOR: 87.5	MOR: 74
**Mean Values**					**79.3**	**92.2**	**83.3**	**91.3**
**SD**					**14.3**	**11.4**	**11.7**	**13.1**
**Clorhexidine**								
Garvey 2007 [30]	12 Pat 10 Cont	CHLOR	ST	CAP	91.6	100	90.8	100
**Biological Agents**								
Chung 2008 [33]	26 Pat 512 Cont	CETUX	CH, DPT	CAP	68.0	98.0	98.4	63.3
Vultaggio 2010 [34]	11 Pat 20 Cont	INFLIX	CH	CAP	27.2	100	71.4	100
Mariotte 2011 [35]	14 Pat 195 Cont	CETUX	CH	ELISA	71.4	82.1	97.6	22.3
Matucci 2013 [36]	30 Pat 50 Cont	INFLIX	CH, ST	CAP	26.0	90.0	66.9	60.9
**Mean Values**					**48.2**	**92.5**	**83.6**	**61.6**
**SD**					**24.9**	**8.2**	**16.8**	**31.7**

Pat: Patients; Cont: Controls; PG: Penicillin G; AX: Amoxicillin; AMP: Ampicillin; CEFU: Cefuroxime; CEFAZ: Cefazolin; CEFs: Cephalosporins; CEFOT: Cefotaxime; CEFT: Ceftriaxone; CEFAC: Cefaclor; PV: Penicillin V; CIPRO: Ciprofloxacin; LOME: Lomefloxacin; NORFLO: Norfloxacin; OFLO: Ofloxacin; PIP: pipemidic acid; RUFL: rufloxacin; PEFL: pefloxacin; NALI: nalidixic acid; MOXI: Moxifloxacin; LEVO: Levofloxacin; MOR: Morphine; SUC: succinylcholine; ALCU: Alcuronium; TMA: trimethylamine; TEA: triethylamine; SUX: sulfamethoxazole; VECU: vecuronium; PANCU: pancuronium; ATRAC: Atracurium; GALLA: gallamine; ROC: Rocuronium; PHO: pholcodine; CHLOR: Clorhexidine; CETUX: Cetuximab; INFLIX: Infliximab; Diag: Diagnostic method; ST: Skin test; DPT: Drug provocation test; CH: Clinical History; RAST: Radioallergosorbent test; CAP: ImmunoCAP-FEIA; SEPH: Sepharose; RIA: Radioimmunoassay; ELISA: Enzyme-linked immunosorbent assay; Sens: sensitivity; Spec: specificity; NPV: negative predictive value; PPV: positive predictive value; and SD: standard deviation.

**Table 2:** Basophil activation tests in immediate reactions to different drugs.

Paper	Patients	Drugs	Diag	Method	Sens (%)	Spec (%)	NPV (%)	PPV (%)
**Betalactam**								
Sanz 2002 [18]	58 Pat 30 Cont	PG, AX, AMP, CEFU, CEFAZ	ST	BAT	50	93.3	49.1	93.5
Torres 2004 [42]	70 Pat 40 Cont	PG, AX, AMP, CEFU, CEFAZ, CEFAC	ST, DPT	BAT	48.6	93	50.8	92.4
Abuaf 2008 [39]	27 Pat 14 Cont	AX, AMP, CEFU	ST	BAT	63	79	52.5	85.2
De Weck 2009 [19]	178 Pat 81 Cont	BP, AX, AMP, CEFs	ST, DPT	BAT	48.3	88.9	43.8	90.5
Torres 2010 [43]	55 Pat 30 Cont	PG, AX, AX-CLV, CLV	ST	BAT	52.7	90	50.9	90.6
Eberlein 2010 [44]	24 Pat 15 Cont	PG, PV, AMP, AX, CEFU	ST	BAT	55	80	52.6	81.5
Sanchez-Morillas 2010 [45]	9 Pat 5 Cont	CLV	ST, DPT	BAT	44.4	100	49.9	100
**Mean Values**					**51.7**	**89.2**	**50.0**	**90.5**
**SD**					**6.0**	**7.5**	**3.0**	**5.9**
**Fluoroquinolones**								
Aranda 2011 [31]	38 Pat 25 Cont	CIPRO, MOXI, LEVO	CH, DPT	BAT	71.1	88	66.7	90.1
Rouzaire 2012 [53]	17 Pat 15 Cont	CIPRO, MOXI, LEVO, OFLOX, LOME, FLUME, NORFLO, PIPEMI	ST, DPT	BAT	76.5	100	78.9	100
Mayorga 2013 [52]	28 Pat 20 Cont	CIPRO, MOXI	CH, DPT	BAT	57.1	90	59.9	88.9
Fernandez 2016 [40]	17 Pat 18 Cont	CIPRO, MOXI	CH, DPT	BAT	52.9	88.9	66.7	81.8
**Mean Values**					**64.4**	**91.7**	**68.1**	**90.2**
**SD**					**11.2**	**5.6**	**7.9**	**7.5**
**NMBAs**								
Abuaf 1999 [51]	21 Pat 29 Cont	SUX, GALLA, VECU, PAN	CH, ST	BAT	64.0	93.0	78.1	86.9
Monneret 2002 [25]	39 Pat 17 Cont	ROC, SUX, ATRAC	CH, ST	BAT	54.0	100	48.6	100
Sudheer 2005 [48]	14 Pat 10 Cont	ROC, ATRAC, SUX, VECU	CH, ST	BAT	78.6	100	76.9	100
Ebo 2006 [46]	14 Pat 8 Cont	ROC	ST	BAT	91.7	100	87.3	100
Kvedariene 2006 [47]	47 Pat 45 Cont	ROC, VECU, ATRA, PAN, SUX	ST, DPT	BAT	36.1	93.3	58.3	84.9
Leysen 2011 [24]	41 Pat 25 Cont	ROC	CH, ST	BAT	80.5	96.0	74.5	97.0
Hagau 2013 [49]	22 Pat 34 Cont	ATRAC, ROC, SUX, PAN	ST	BAT	68.2	100	82.9	100
Uyttebroek 2014 [50]	8 Pat 7 Cont	ATRA	ST	BAT	62.5	100	70.0	100
**Mean Values**					**66.9**	**97.8**	**72.1**	**96.1**
**SD**					**17.2**	**3.2**	**12.9**	**6.4**
**Pyrazolones**								
Gamboa 2003 [55]	26 Pat 30 Cont	META	ST, DPT	BAT	42.3	100	66.7	100
Gomez 2009 [56]	51 Pat 56 Cont	META	CH, ST, DPT	BAT	54.9	85.7	65.1	79.6
Hagau 2013 [54]	20 Pat 10 Cont	DIP	ST	BAT	65.0	100	58.8	100
**Mean Values**					**54.1**	**95.2**	**63.5**	**93.2**
**SD**					**11.4**	**8.3**	**4.2**	**11.8**
**Radio Contrast Media**								
Pinnobphun 2011 [57]	26 Pat 43 Cont	IOXIT, IOPR, IOPA, IOH, IOB	CH, ST	BAT	57.7	97.7	79.3	93.8
Salas 2013 [58]	8 Pat 20 Cont	IOD, IOH, IOM, IOB	ST, DPT	BAT	62.5	100	86.9	100
**Mean Values**					**60.1**	**98.9**	**83.1**	**96.9**
**SD**					**3.4**	**1.6**	**5.4**	**4.4**

Pat: Patients; Cont: Controls; PG: Penicillin G; AX: Amoxicillin; AMP: Ampicillin; CEFU: Cefuroxime; CEFAZ: Cefazolin; CEFAC: Cefaclor; CEFs: Cephalosporins; CLV: Clavulanic acid; CIPRO: Ciprofloxacin; MOXI: Moxifloxacin; LEVO: Levofloxacin; OFLO: Ofloxacin; LOME: Lomefloxacin; FLUME: Flumequin; NORFLO: Norfloxacin; PIPEMI: Pipedimic acid; SUX: Suxamethonium; GALLA: gallamine; VECU: Vecuronium; PAN: Pancuronium; ROC: Rocuronium; ATRAC: Atracurium; META: Metamizole; DIP: Dipirone; IOXIT: Ioxithalamate; IOPR: Iopromide; IOPA: Iopamidol; IOH: Iohexol; IOB: Iobbitrol; IOD: Iodixanol; IOM: Iomeprol; Diag: Diagnostic method; CH: Clinical History; ST: Skin test; DPT: Drug provocation test; BAT: Basophil activation test; Sens: sensitivity; Spec: specificity; NPV: negative predictive value; PPV: positive predictive value; and SD: standard deviation.

**Table 3:** In vitro tests in non-immediate reactions to different drugs.

Paper	Patients	Clinical Entity	Drugs	Diag	Method	Sens (%)	Spec (%)	NPV (%)	PPV (%)
**LTT**									
Roujeau 1985 [75]	12 Pats 8 Cont	TEN	Ant-Con, NSAID	CH	Thymidine	44.0	63.0	42.9	64.1
Nyfeler 1997 [70]	100 Pat 102 Cont	ND	BLs	CH, ST	Thymidine	74.4	85.0	77.2	82.9
Orasch 1999 [69]	10 Pat 6 Cont	URT/ANG, EXANT	LA	CH, ST	Thymidine	60.0	100	60.0	100
Schnyder 2000 [66]	12 Pat 6 Cont	EXANT	BLs	CH	Thymidine	83.3	100	74.9	100
Luque 2001 [71]	19 Pat 28 Cont	URT, EXANT	BLs	ST, DPT	Thymidine	57.9	92.8	76.5	84.5
Hari 2001 [72]	21 Pat 16 Cont	MPE, BULL-EXANT, URT	Ant-Con, Ant-hyp, others	CH	Thymidine	66.6	93.8	68.2	93.4
Sachs 2002 [74]	10 Pat 10 Cont	MPE, AGEP, TEN	BLs, Ant-Con	CH, ST	Thymidine	75.0	100	80.0	100
Rodriguez-Pena 2006 [68]	9 Pat 8 Cont	MPE	BLs	CH, ST	Thymidine	22.2	100	53.3	100
					+ DC	88.8	100	88.8	100
Suzuki 2008 [73]	69 Pat 50 Cont	BULL- EXANT, DILI	Ant-tub	CH, DPT	Thymidine	28.9	90.7	48.03	81.1
Rozieres 2009 [67]	22 Pat 11 Cont	MPE	BLs	ST	Thymidine	68.2	100	61.1	100
Whitaker 2011 [65]	28 Pat	URT/ANG, MPE, others	BLs	CH	Thymidine	64.3	ND	ND	ND
Polak 2013 [77]	43 Pat 14 Cont	MPE, DRESS, TEN, FDE, ECZ	Various	CH	Thymidine	25.0	95.1	29.2	94.0
Porebski 2013 [76]	15 Pat 18 Cont	SJS/TEN	Ant-Con	CH	Thymidine	26.6	100	62.0	100
**Mean Values**						**56.1**	**93.9**	**63.2**	**92.3**
**SD**						**22.7**	**10.4**	**16.8**	**11.1**
**ELISpot**									
Rozieres 2009 [67]	22 Pat 11 Cont	MPE	BLs	ST	IFN-γ	90.9	100	84.6	100
Polak 2013 [77]	43 Pat 14 Cont	MPE, DRESS, TEN, FDE, ECZ	Various	CH	IFN-γ, IL-4	IFNγ: 50.0 IL-4: 50.0	IFNγ: 82.9 IL-4: 92.0	IFNγ: 35.1 IL-4: 37.5	IFNγ:90.0 IL-4: 95.0
Porebski 2013 [76]	15 Pat 18 Cont	SJS/TEN	Ant-Con	CH	GranzymeB	33.3	98.0	63.8	93.3
Tanvarasethee 2013 [85]	25 Pat 20 Cont	MPE	BLs	CH	IFN-γ, IL-5	40.0	100	57.1	100
Klaewsongkram 2016 [86]	24 Pat 21 Cont	DRESS, SJS/TEN	Allop	CH	IFN-γ	79.2	95.2	80.0	95.0
Kato 2017 [87]	16 Pat 3 Cont	EXANT, DRESS, TEN, SJS	Ant-Con	CH	IFN-γ	85.0	100	55.5	100
**Mean Values**						**61.2**	**98.6**	**59.1**	**96.2**
**SD**						**23.3**	**6.3**	**19.0**	**3.9**
**ELISA+Flow Cytomety**									
Sachs 2002 [74]	10 Pat 10 Cont	MPE, AGEP, TEN	BLs, Ant-Con	CH, ST	IL-5, IFN-γ, IL-10	IL5: 91.6 IFNγ: 36.4 IL10: 50.0	IL5: 100 IFNγ: 60.0 IL10: 100	IL5: 92.3 IFNγ: 48.5 IL10: 66.7	IL5: 100 IFNγ: 47.6 IL10: 100
Khalil 2008 [94]	15 Pat 12 Cont	URT/ANG, MPE	BLs	ST	IL-2, IL-5, IFN-γ	IL-2: 86.7 IL-5: 100 IFNγ: 78.5	IL-2: 100 IL-5: 62.5 IFNγ: 90.0	IL-2: 85.7 IL-5: 100 IFNγ: 77.1	IL-2: 100 IL-5: 76.9 IFNγ: 90.8
Halevy 2008 [95]	12 Pat 11 Cont	VASC, URT, MPE, TEN, FDE, Others	Various	CH	IFN-γ	80.0	62.0	74.0	70.0
Martin 2010 [93]	19 Pat 10 Cont	URT, MPE, TEN, others	BLs, Ant-Con, RCM, others	CH	IL-2, IL-5, IFN-γ	IL2: 43.0 IL5: 43.0 IFNγ: 57.0	100	IL2: 48.0 IL5: 48.0 IFNγ: 55.0	100
**Mean Values**						**66.6**	**87.5**	**69.5**	**88.5**
**SD**						**23.2**	**18.2**	**19.4**	**18.1**

Pat: Patients; Cont: Controls; TEN: Toxic epidermal necrolysis; ND: Not determined; URT: Urticaria; ANG: Angioedema; EXANT: Exanthema; MPE: Maculopapular exanthema; BULL-EXANT: Bullous Exanthema; AGEP: Acute generalized exanthematic pustulosis; DILI: Drug induced liver injury; DRESS: Drug rash with eosinophilia and systemic symptoms; SJS: Stevens–Jonhson Syndrome; FDE: Fixed drug eruption; ECZ: Eczema; VASC: Vasculitis; Ant-Con: Anti-convulsant; NSAID: non-steroidal anti-inflammatory drugs; BLs: Betalactams; LA: Local Anesthetics; Ant-hyp: Anti-hyperthensive; Ant-tub: Anti-tuberculosis; Allop: Allopurinol; Diag: diagnostic method; CH: Clinical History; ST: Skin test; DPT: Drug provocation test; DC: Dendritic cells; Sens: Sensitivity; Spec: Specificity; NPV: Negative predictive value; PPV: Positive predictive value.

## Abbreviations

DHR	Drug Hypersensitivity Reactions
SJS	Steven–Johnson Syndrome
TEN	Toxic Epidermal Necrolysis
IDHR	Immediate DH
sIgE	Specific IgE
NIDHR	Non-Immediate DHR
STs	Skin Tests
DPT	Drug Provocation Tests
BLs	β-Lactam
RCM	Radio Contrast Media
BAT	Basophil Activation Test
LTT	Lymphocyte Transformation Tests
ELISpot	Enzyme-Linked Immunosorbent Spot Assay
ELISA	Enzyme-Linked Immunosorbent Assay
PPV	Positive Predictive Value
NPV	Negative Predictive Value
RAST	Radioallergosorbent Test
NMBAs	Neuromuscular Blocking Agents
FQs	Fluoroquinolones
NSAIDs	Non-Steroidal Anti-Inflammatory Drugs
CFSE	Carboxyfluorescein Diacetate Succinimidyl Ester
